# Barriers to participation in Iran’s Integrated Geriatric Care Program: A qualitative study

**DOI:** 10.1371/journal.pone.0315034

**Published:** 2025-02-14

**Authors:** Neda Sadat Nazaripanah, Haidar Nadrian, Vali Bahrevar, Elham Lotfalinezhad, Mina Hashemiparast

**Affiliations:** 1 Aging Research Institute, Tabriz University of Medical Sciences, Tabriz, Iran; 2 Department of Health Education and Promotion, Tabriz University of Medical Sciences, Tabriz, Iran; 3 Social Determinants of Health Research Center, Tabriz University of Medical Sciences, Tabriz, Iran; 4 Department of Aging, University of Social Welfare and Rehabilitation Sciences, Tehran, Iran; 5 Department of Psychiatry and Community Health Nursing, School of Nursing and Midwifery, Golestan University of Medical Sciences, Gorgan, Golestan, Iran; 6 Social Determinants of Health Research Center, Health and Metabolic Disease Research Institute, Zanjan University of Medical Sciences, Zanjan, Iran; 7 Department of Health Education and Promotion, School of Public Health, Zanjan University of Medical Sciences, Zanjan, Iran; Shahid Beheshti University of Medical Sciences School of Dentistry, ISLAMIC REPUBLIC OF IRAN

## Abstract

**Background:**

A significant number of older adults still have unfulfilled needs related to healthcare and social services. Policymakers and health administrators can turn this challenge into a golden opportunity through proper planning and policymaking. This study aimed to identify barriers to participation in Iran’s “Integrated Geriatric Care Program (IGCP)”.

**Methods:**

A qualitative study with conventional content analysis approach was conducted in Yazd City, Iran, from January to March 2023. A purposive sampling method with maximum variation was used. Nineteen healthcare providers and community-dwelling older adults participated in the study. Data were collected using semi-structured in-depth interviews until data saturation and were concurrently analyzed.

**Results:**

Nine older persons (3 women and 6 men) with a Mean±SD age of 71.33±5.75 and ten healthcare providers (6 women and 4 men) with a Mean±SD age of 38.2±9.16 participated in the study. Two main categories, with ten sub-categories, emerged from the data analysis. The main categories were “personal challenges of older adults” and “structural defects of Integrated Geriatric Care Program”.

**Conclusion:**

The participation of community-dwelling older adults in the Iranian IGCP is constrained by a combination of personal and structural factors. Our findings seem to be informative for health policymakers and health practitioners who aim to enhance the quality and quantity of healthcare services for older adults in developing communities.

## Introduction

According to literature [1], the older adult population will surpass the younger population globally by 2040 [2]. This is while population aging is a general challenge for all nations [3, 4]. Despite being a priority for governments and policymakers in responding to the needs of older adults, the needs of older adults are frequently overlooked in developing countries [5].

Older people are recognized as vulnerable groups, and it is essential to provide them with healthcare services [6, 7]. The care needs of older adults are multidimensional, diverse, complex, and often time-consuming [8]. Approximately 80 percent of older Americans have at least one chronic disease [9] and 86% of all healthcare costs are related to such diseases, which is even greater when considering the global data [10]. To maintain the health of older adults and manage such conditions, European countries have offered various care models for older people, such as preventive care services, regular physician visits, assessment and screening of chronic diseases, etc [11]. In Iran, as a developing country, the Ministry of Health and Medical Education has implemented the Integrated Geriatric Care Program (IGCP) across all public healthcare centers catering to individuals aged 60 and above [12]. Nevertheless, a considerable portion of the older population in Iran continues to have unmet needs in terms of healthcare and social services [13], and thus the participation rate of Iranian older adults in this program has reported to be low [14]. Evidence suggest that the current healthcare services are not suitable for meeting the needs of older adults in Iran, and emphasized the need for further investigations [15].

Several previous studies in various countries have highlighted many possible reasons for lack of participation of older adults in obtaining healthcare services. Examples of the reasons are reported to be psychological and physical issues [16], concerns about affordability and poverty [17], type of living arrangement [18], limited access to services, poor policymaking, lack of inadequate technical infrastructure, and lack of integrity and coordination of healthcare services [15]. However, a limit number of studies has investigated the barriers and challenges in an in-depth manner, particularly from the perspectives of both community-dwelling older adults and healthcare providers. Hence, this study aimed to explore the barriers associated with the provision and reception of the IGCP as perceived by both community-dwelling older adults and healthcare providers in Yazd City, Iran.

## Materials and methods

### Study design and participants

This was a qualitative study with a conventional content analysis approach, performed from January to March 2023, to explore the challenges of providing and receiving IGCP in Yazd City, Iran. Participants were healthcare providers and older persons referring to the urban healthcare centers. We chose to conduct the study qualitatively, as we believed that the barriers and challenges of older adult^,^s participation in geriatric care programs are understudied. Also, the conventional content analysis approach was considered to be suitable for our study, as it will allow us to interpret the data well (through latent coding), with the hope to offer more than a surface description of the barriers.

### Study setting

Imamshahr Healthcare Center (IHC) in Yazd City, a historical city in central Iran, was established in 1987 to provide the local community with healthcare services. The total population covered by the center includes 16,204 people, of which 18.95 percent (3,071 people) are older adults (60 years and older). The healthcare services provided in this center are general physician visits, primary healthcare services (PHC), midwifery, nutrition, and mental healthcare services. IGCP is another set of healthcare services that have been provided for older adults in the center since about eight years ago.

### Sampling

A purposeful sampling method was used to recruit participants from two distinct groups: healthcare providers and older adults. We used maximum variation with the hope to construct a holistic understanding of the barriers of participation in IGCP. To achieve maximum variation practicaly, we tried to identify key aspects of variations in the participants, and then invited the cases that vary from each other as much as possible to particpate in the study. For healthcare providers, maximum variation was ensured in terms of gender, work experience, and position. For older adults, participants were selected based on diversity in gender, age, health status, and the amount of healthcare services use. The inclusion criteria for older adults were as follow: being 60 years of age and older, having electronic health records in healthcare center, using geriatric care services, and willingness to participate in this study. For healthcare providers, the inclusion criteria were having work experience of at least two years, familiarity and involvement in IGCP, and willingness to participate in this study. Accordingly, nineteen individuals participated in the study, and their demographic information is presented in Table 1.

**Table 1:** Profile of participants (n = 19).

Participants code	Sex	Age (Year)	Position (Field of study)	Work experience (Year)
1	Male	72	Retired	-
2	Male	65	Retired	-
3	Male	78	Retired	-
4	Male	68	Retired	-
5	Male	63	Retired	-
6	Female	80	Housewife	-
7	Female	66	Housewife	-
8	Female	76	Housewife	-
9	Male	74	Retired	-
10	Female	52	Healthcare provider (Public health)	17
11	Male	31	Healthcare provider (Physician)	3
12	Female	45	Healthcare provider (Public health)	24
13	Female	34	Healthcare provider (Midwifery)	8
14	Male	27	Healthcare provider (Psychology)	4
15	Female	30	Healthcare provider (Public health)	8
16	Male	34	Healthcare provider (Nutrition)	10
17	Male	50	Healthcare provider (Public health)	27
18	Female	49	Healthcare provider (Public health)	30
19	Female	30	Healthcare provider (Physician)	2

### Data collection and analysis

Data were collected using semi-structured individual interviews. The interviews were conducted using an interview guide with open-ended questions. To design the set of questions in the interview guide, we firstly considered the research question to determine the broad area that our interview may cover. To do so, the team of research in one session examined what we want to hear from the participants. This helped us in perceiving the general direction of the questions, and in listing the topics of the questions in detail. Eventually, we wrote the interview questions, and finalized the interview guide (Table 2).

**Table 2:** Semi-structured interview guide.

	**Interview questions**	
Asked from older adults	1	What barriers and/or challenges may impede you from receiving IGCP services?
	2	How do you ubderestand and describe the program services?
Asked from healthcare providers	1	What are the barriers and challenges of providing older adults with IGCP services?
	2	What are the barriers and facilitators of program implementation?
	3	How is the progress of health indicators?
	4	What are the reasons for rejection of the program?
	5	How do you describe the adoption of the program services by older adults?
	6	What is the gap in the current service delivery?

The interview usually began with a main research question and ended with a series of probing follow-up questions such as ‘would you please detail your explanation?’ and ‘would you explain more, please?’, according to the responses of the participants.

The time and place of the interviews were determined by mutual agreement between interviewee and the interviewer. Hence, the place of interview was either the health center or the interviewer work place. Each interview lasted 30 to 60 minutes. The interviews were continued until data saturation, where the researcher began to hear the same comments again and again and no new theme or idea emerged [19].

Data analysis was conducted simultaneously with data collection. The recorded interviews were transcribed verbatim before starting the next interview. The structure of themes was optimized using the conventional content analysis approach [20]. Accordingly, the data were broken down into meaning units that were extracted from the statements and labeled with conceptual names (codes). After this open coding, the codes were compared based on similarities and differences and grouped into categories. Each subcategory with similar meaning was grouped together as categories, and the categories are then grouped as main categories. MAXQDA-32 software was used to manage the textual data during the coding process.

### Ethical considerations

This study is approved by the Research Ethics Committee at Tabriz University of Medical Sciences (IR.TBZMED.REC.1401.100). All participants were given a form containing the purpose of this project and written informed consent. Participants’ identities and responses were kept confidential, anonymous, and only accessible to the research team.

### Trustworthiness

The criteria suggested by Guba and Lincoln was applied to evaluate the credibility of the data [21]. Peer debriefing was conducted to indicate our position toward data and analysis. Also, the research team checked the interview data and findings at each step of the study. Moreover, analytic categories, interpretations, and conclusions were tested using member checks [22]. All steps followed in the research process were documented by the researchers to provide auditability of the data [23]. The guideline of consolidated criteria for reporting qualitative research (COREQ) was used while providing the manuscript [24].

## Results

Nine older persons (3 women and 6 men) with a Mean±SD age of 71.33±5.75 and ten healthcare providers (6 women and 4 men) with a Mean±SD age of 38.2±9.16 participated in the study. In this study, we aimed to explore the barriers associated with the provision and reception of the IGCP as perceived by both community-dwelling older adults and healthcare providers. According to data analysis, 173 initial codes were extracted, which were summarized in 10 sub-categories and two main categories. The two main categories (*personal challenges of older adults* and *structural defects of IGCP*) and their associated sub-categories (Table 3) are presented as follows:

**Table 3:** Extracted themes and sub-themes.

Themes	Sub-themes
Personal challenges of older adults	The lack of information about healthcare services Inhibitory mental beliefs Lack of motivation to participate in IGCP^a^ Inability to visit the healthcare center Lack of feeling of need to use healthcare services
Structural defects of IGCP	The lack of resources and facilities (the shortage of employees; lack of suitable physical space and sufficient medical equipment; lack of sufficient budget allocation) Not being free of charge for some healthcare services The defect of registration system High workload of healthcare providers Lack of skillful healthcare providers

^a^IGCP: Integrated Geriatric Care Program

### 1. Personal challenges of older adults

This category presented the personal challenges of older adults to participate in healthcare center, which had five sub-categories as described below:

#### 1.1. The lack of information about healthcare services

This concept refers to the lack of widespread promotion of the activities provided by the healthcare center in primary and community-based care for older individuals. According to participant statements, some older adults were unaware of these services offered at the healthcare center:

*“…I’m not aware of the older adult care program you’re referring to, and I’m unsure about the services offered at the healthcare center. However, if there is a care program available, I would like to participate and undergo assessment.*” *(p1)*

Accordingly, some participants believed that information, awareness, and advertising about healthcare services would lead to an increase in the participation of older adults in IGCP. In this regard, one of the healthcare providers explained:

*“…There is no comprehensive information and advertising for services provided by healthcare centers at the national level or on social media like radio and television. If extensive advertising efforts were implemented, the elderly population would likely turn to healthcare centers to access care services. The fact is that there is no advertising here*.” *(p13)*

#### 1.2. Inhibitory mental beliefs

Participants admitted that some beliefs are barriers to accessing IGCP services at the healthcare center, potentially influencing individuals’ decisions regarding adopting a healthy lifestyle. For example, one of the healthcare providers stated:

*“…Sometimes healthcare providers offer recommendations yet some older adults persist in adhering to their own beliefs and lifestyles, refusing to accept or criticizing the advice provided. For instance, when we suggested the use of liquid oil instead of solid oil due to concerns about buildup and blockage in blood vessels, an older person countered by asserting that liquid oils are adulterated and insisted that solid oils are preferable*.” *(p12)*

*“…Some older adults believe that because they do not pay for IGCP services therefore the quality of services is low*. *Moreover*, *they stated that the healthcare provider may not have enough knowledge and skills*, *so they do not refer to the healthcare center*.” *(p17)*

#### 1.3. Lack of motivation to participate in IGCP

According to the participants, there is a low level of motivation among stakeholders to participate in IGCP. Moreover, Furthermore, participants attributed this issue to the perceived failure of the IGCP to fulfill the demands of older adults, such as providing medication, specialized care, and conducting necessary tests:

*“…the majority of older individuals expect us to provide medication tailored to their specific conditions. Additionally, they request the availability of specialist physicians at the healthcare center*.” *(p11)*

Additionally, some participants highlighted that the ineffectiveness of services and the mismatch between health recommendations and the needs of older individuals result in a lack of motivation and desire to visit healthcare centers for services. According to these participants, the services offered are often repetitive and primarily consist of counseling rather than treatment, which older adults perceive as not very effective:

*“…The IGCP hasn’t influenced my lifestyle or health. Healthcare providers aren’t offering new recommendations. The services they provide are mainly advisory, and the education is often repetitive. Their recommendations don’t align with my needs, and I find them difficult to follow*.” *(p2)*

#### 1.4. Inability to visit the healthcare center

The participants noted that personal challenges such as disabilities, illnesses, both physical and psychological issues, limited access to transportation, and adverse weather conditions act as barriers for older adults in attending healthcare centers. The statements from the participants further supported this perspective:

*“…The health status of older individuals differs significantly from other age groups. They often contend with issues such as knee and foot pain, and some may be living with disabilities. For instance, we encountered several individuals grappling with Parkinson’s disease, making it challenging for them to come here*.” *(p11)*

#### 1.5. Lack of feeling of need to use healthcare services

Participants acknowledged that certain factors such as the absence of illness, access to domestic health facilities and equipment, and availability of specialized care have contributed to a reduced demand for the IGCP among older adults:

*“…Some older individuals may possess education or have families with adequate expertise. They may have access to medical equipment such as a sphygmomanometer for measuring blood pressure or a glucometer for monitoring blood sugar levels at home. Consequently, they may find it unnecessary to visit healthcare center*.” *(p19)*

### 2. Structural defects of IGCP

This category included five sub-categories that described structural defects of the IGCP services in healthcare center:

#### 2.1. The lack of resources and facilities

The scarcity of resources and facilities, including insufficient staffing, inadequate physical space, a shortage of medical equipment, and limited budget allocations, were highlighted as significant challenges. Participants’ statements pointed to a deficiency in skilled human resources necessary for effectively implementing the IGCP within healthcare centers:

*“…The number of healthcare providers is not proportional to clients. I believe that experts and graduates in the field of geriatrics, and gerontology are needed to carry out geriatric care programs. A person who has more knowledge in the field of aging and can assess older adults better. Following this, the quality of providing care services will increase*.” *(p16)*

Participants highlighted that the absence of a suitable environment, such as a dedicated assessment room tailored to the needs of older adults, has resulted in dissatisfaction among them. Additionally, the scarcity of necessary equipment, like a glucometer for blood sugar measurement, was mentioned by participants, as exemplified in one participant’s statement:

*“…The healthcare center is consistently bustling and loud often filled with the presence of children, which can be quite bothersome due to excessive noise. I believe it would be beneficial to allocate a private room specifically for assessments of older adults to ensure a quieter and more conducive environment. Additionally, the healthcare center’s equipment, such as glucometers, is insufficient to meet the demands effectively*.” *(p7)*

#### 2.2. Not being free of charge for some healthcare services

This concept emphasize that while some services may be free, there are still costs involved for certain aspects of healthcare. Many participants expressed that the services offered to older adults are not entirely free. Consequently, they observed a lack of participation among older adults in the IGCP:

*"…Some older patients expect all the services of the healthcare center including physician’s visits, tests, and medications, to be provided to them for free. However, their demands are not met by IGCP. In general, most older adults question why they should choose this healthcare center, given its lack of special services. Even a physician’s visit is not free. They prefer to refer to other centers that offer both free and specialized services*." *(p10)*

#### 2.3. The defect of registration system

The participants highlighted technical problems within the registration electronic system as a significant barrier to delivering care services to older adults. Some healthcare providers emphasized that a comprehensive and efficient registration system could serve as the foundation for accurate assessments and increased engagement of older individuals:

*"…The registration electronic system is not a more comprehensive approach. If a revision is made to the system, the delivery of care services will be better. For example, there have been many instances where the system suddenly disconnected, resulting in the complete deletion of the questions I asked. I must log in again and repeat the process. These problems are troublesome and tedious. As you know, elderly clients cannot afford to wait too long*." *(p15)*

#### 2.4. High workload of healthcare providers

Most of the participants reported that providing geriatric care to an older person requires more time than other age groups. Factors such as the multitasking of personnel, the large number of variety clients and care programs, busy work, and time constraints have caused difficulties for healthcare providers in allocating adequate time to providing care services to older adults, and are often in a hurry. They believe that these problems have affected the quality and quantity of IGCP delivery. This belief was illustrated by one of the participants’ statement:

*“…Each healthcare provider is responsible for performing all care programs for individuals across all age groups within the population served by the center. At times, the overcrowding of the center compels healthcare providers to rush through IGCP delivery in order to attend to the next client*.” *(p16)*

#### 2.5. Lack of skillful healthcare providers

Participants asserted that barriers to providing IGCP include insufficient knowledge among staff in the field of geriatric care, carelessness in providing care, low awareness, and negative attitudes of staff towards older adults, as well as neglect of their needs. This perspective was reinforced by the following statements:

*“…Policymakers and practitioners of IGCP should keep in mind that healthcare providers need more training in the field of geriatrics. But in my opinion, among the age groups, there is the most negligence in caring for the elderly. This performance is probably due to their low awareness and attitude toward older adults*.” *(p15)*

*“…The online registration system is complex*. *We do not have enough information about it and we have not seen training*. *We learned the operation of the application automatically*. *If the staff are given sufficient training and information*, *it will be effective in the quality and quantity of IGCP*.” *(p18)*

## Discussion

This study aimed to identify barriers of community-dwelling older adults’ participation in the Iranian IGCP. Both older adults and healthcare providers have expressed concerns about insufficient advertising regarding free-of-charge care services for older adults in the community. As a result of insufficient advertising, many older adults may be unaware of the existence of free-of-charge care services available to them. Consequently, they may not take advantage of these services, leading to underutilization or low participation rates. Previous research suggests that a lack of information about healthcare services for older adults can result in their inability to access suitable services, thereby failing to meet their healthcare needs [25–27].

On the other hand, according to a life course approach to health literacy, older adults with lower health literacy compared to others, may struggle to seek and access comprehensive information about available healthcare services in the community through traditional media channels [28, 29]. Thus, creating age-friendly media platforms could offer an effective opportunity for community-dwelling older adults to enhance their awareness of these care services within their community [30].

Inhibiting mental beliefs was identified as one of the most important obstacles. Additionally, older adults were less likely to change their current diet, which can be associated with continuity theory in aging. According to this theory, older adults tend to sustain their past values, beliefs and behaviours [31]. In the current study, older adult participants expressed that saturated oil is better compared to liquid oil because there is very common fraud in the production of liquid oil. Previous studies indicated that if health providers can establish a strong relationship in which older adults’ concerns are addressed based on their age and abilities, the older adults will be able to perceive and pursue health providers’ recommendations [32, 33].

Older adults expressed that due to free healthcare services, and low level of health professionals’ knowledge, providing care services for older adults was inadequate, just focused on checking blood pressure and blood sugar. Thus, this approach declines the level of older adults’ contributions to attending healthcare center because of ineffectiveness of healthcare services and the failure to meet the expectations of older adults. The higher quality of healthcare service and clients’ satisfaction regarding care services can enhance the credibility of physician-patient communication [34].

Moreover, this study shows that healthcare providers did not have any motivation to make a recommendation to older adults regarding healthy eating and physical activity because healthcare providers believed that these recommendations were not based on their physical and financial condition and that this information was merely recorded in the e-medical file. Previous studies suggest that the person-centered recommendation approach is more likely to increase older adults’ motivation to adopt a healthy lifestyle. In other words, taking advantage of individualized, specific, and achievable goals may promote the likelihood of healthy behavior change in the future [32, 35, 36].

Notably, this study reveals that not feeling the need to use healthcare services due to the availability of health equipment (e.g. Digital sphygmomanometer) at home can influence older adults’ attendance to healthcare centers. However, regular physical and mental health check-ups for older adults may give healthcare providers and family physicians a chance to identify any abnormalities or symptoms of disease at early stage and provide an opportunity for successful treatments [37], which in turn reduces the health expenditures in the countries [38].

The lack of ability of older adults to attend health center was another challenge that led to the inaccessibility of care services. The older adults stated that they were not able to attend the health center because of their disability, transportation problems, bad weather conditions and lack of social support. Some countries apply different care models to deliver healthcare services for older adults. The National Health Care Program for the Elderly in India, for instance, trained healthcare providers to provide primary health care services at older adults’ homes so that all older adults with various needs can benefit from these care services [39].

Participants in this study noted that having sufficient medical equipment (e.g. a glucometer) and geriatric specialists in healthcare centers is critical for enhancing the involvement of older individuals in seeking healthcare services. This finding is supported by previous research indicating the scarcity of qualified professionals and healthcare technologies lead to obstacles in terms of accessing health services for older adults [40]. Consistent with these findings, the challenges concerning the aging population in the health system in Iran are insufficient expertise and specialized knowledge and the incapacity to allocate an appropriate budget to care for the aging population [41]. According to participants’ reports, the complexity and time-intensive aspect of providing preventative healthcare for older persons in the multi-specialty field of geriatrics is an additional obstacle that health providers confront [42, 43]. Prior studies indicate that due to staff shortages and specialized nursing staff for older adults, the healthcare providers experience an extensive workload, which results in decreasing the quality of healthcare [15, 44].

On the other hand, if a client needs to be visited by physician more than once at a health center, the client has to spend out-of-pocket for the next medical examination. Therefore, older adults do not have any tendency to refer to these centers. Instead, they attend centers in which offer free and more specific care services for older clients with multiple chronic diseases. This finding is in agreement with Lyttle’s (2010) study, which indicated that providing free care services can enhance older adults’ participation in receiving care services [45]. In Iran, approximately, 25 percent of older adults do not have insurance coverage and existing insurance plans do not cover the majority of care services for older adults [46]. Based on the United Nations Population Fund report, because of a lack of adequate insurance coverage, care services are not accepted by clients [47]. Participants in the current study noted that technical issues such as limited access to patients’ medical records and frequent internet disconnects in the e-registration system hinder the provision of care services to older people. The results align with previous studies that revealed the network’s signal and integrity of health data input play a critical role [48, 49]. One solution to this challenge, which was proposed by some healthcare practitioners, would be, a thorough and efficient e-registration system might serve as the foundation for precise evaluation and increased acceptance of older individuals [50]. In Australia, healthcare personnel provide necessary care for older adults through the My Aged Care telephone service [51]. Similarly, personal and social support networks for people with chronic diseases have recently been developed, which provide a list of resources tailored to client’s needs and interests using online navigation tools [52].

### Limitations

The study spanned a considerable duration primarily due to the time-consuming nature of conducting interviews with older adults. Additionally, due to the heightened workload of healthcare providers, scheduling interviews with them posed challenges, necessitating some meetings to be conducted outside regular office hours. Furthermore, the cooperation from elderly women was comparatively lower than that from men.

### Study recommendations

Although the current study has tried to capture an authentic perspective on the barriers and challenges surrounding the providing and receiving of IGCP from the viewpoint of key stakeholders, it acknowledges its limitations in fully uncovering all relevant facts and obstacles associated with this program. Therefore, it is recommended to conduct further investigations to explore viewpoints of policymakers and managers of concerning this program. Moreover, it is advisable for researchers to prioritize these obstacles in forthcoming studies and devise intervention strategies based on their significance and potential for change.

## Conclusion

This study provides evidence that the participation of community-dwelling older adults in the Iranian IGCP is constrained by a combination of personal and structural factors. Health policymakers in developing countries, and Iran, are recommended to improve geriatric healthcare services through adopting a comprehensive, continuous, participatory, supportive, and active approach. Future research should be focused on how such an approach should be designed, planned, and applied in the healthcare setting of developing countries. Our findings seem to be informative for health policymakers and health practitioners who aim to enhance the quality and quantity of healthcare services for older adults in developing communities.
